# The Woman Who Lives without Eating

**Published:** 1859-04

**Authors:** 


### "THE WOMAN WHO LIVES WITHOUT EATING."

In the present number of our Journal will be found a report of two rare cases—one taken from the Boston "Medical and Surgical Journal" and the other from the "London Lancet."

The one from the former Journal, familiarly known as "The woman who lives without eating," has for several months past made a great noise both in and out of the profession.

We have from the first believed that there was practiced, in this remarkable case, great deception, and feel that the case above alluded to from the "London Lancet" under the caption of "The Dead Alive" confirms our first impressions.

Below will be found an editorial from the "Boston Journal" relative to the case:

"*The Woman who Lives on Nothing.*"

"Under this caption, we to-day publish an article furnished by a highly respectable physician of the State of New York; but take occasion to give our opinion as to certain of the statements, in the truth of which we have no confidence whatever. We must not be understood as impugning the veracity of the reporter of the case; and, in respect to the phenomena which he personally observed, we would say that we do not doubt them at all. Such phenomena are not unusual, however, although in different cases there are various manifestations. It is only in regard to statements which were made to the reporter, that we demur—as all medical men do—or should—to the relations and *dicta* of friends and bystanders. Few, if any, reports on medical and surgical points are worthy of trust, unless furnished by a physician or surgeon who has seen everything of which he writes or speaks. Now, Dr. Whiting was not present with this patient for the entire "two years" during which, he informs us in his paper, "she has had no dejections from the bowels, nor any secretion of urine;" and he ought to know that this assertion implies a physical impossibility, and is an absurdity. Nor is it any more within the bounds of belief, that "for the space of two years from last June, she [the patient] has not taken any food of any kind or description; nor that she has swallowed any liquids for two years from this last February. We repeat, neither of these assertions are at all credible, nor are such things even possible. It is only a day or two since we read, with great interest, in the *London Lancet*, an account, very ably drawn up, explaining the astounding phenomena manifested by a young girl laboring under the severest and most melancholy form of hysteria. The ingenuity displayed by this patient in deceiving those who closely watched her in order to detect her in taking food and drinks, and in urinating, all which, *seemingly*, she did not do—was perfectly marvellous; yet finally she was caught, at midnight, in the act of eating; and linen recently soaked in urine was found in a closet near at hand, but not previously examined. This account may be read in a recent number of the *Lancet*, under the caption, "The Dead Alive."

Thus our correspondent will see—and we conclude he has long known—that apparently inscrutable mystery, in this class of cases, is often at last unravelled; although only after tedious and untiring efforts. We fearlessly express our belief that such *espionage*, properly established and duly maintained over the patient *he visited only once*, would reveal enough to make him change his present inclination "to call it no fraud." Hysteria gives rise to a wonderful series of performances; and we are ready to credit almost anything but the grave averment that a human being can exist two whole years without taking a particle of food or of drinks, and without having any fæcal discharge or renal secretion and flow. The one set of phenomena, it is true, have such a dependence upon the other, that *if the first be proved*, the second—so far at least as the fæcal evacuation is concerned—might be considered a consequence. But *prove* the first!

